# A case of marginal zone B cell lymphoma mimicking IgG4-related dacryoadenitis and sialoadenitis

**DOI:** 10.1186/s12957-015-0459-z

**Published:** 2015-02-21

**Authors:** Miho Ohta, Masafumi Moriyama, Yuichi Goto, Shintaro Kawano, Akihiko Tanaka, Takashi Maehara, Sachiko Furukawa, Jun-Nosuke Hayashida, Tamotsu Kiyoshima, Mayumi Shimizu, Yojiro Arinobu, Seiji Nakamura

**Affiliations:** Section of Oral and Maxillofacial Oncology, Division of Maxillofacial Diagnostic and Surgical Sciences, Faculty of Dental Science, Kyushu University, 3-1-1 Maidashi, Higashi-ku, Fukuoka, 812-8582 Japan; Laboratory of Oral Pathology, Division of Maxillofacial Diagnostic and Surgical Sciences, Faculty of Dental Science, Kyushu University, Higashi-ku, Fukuoka, Japan; Department of Oral and Maxillofacial Radiology, Kyushu University Hospital, Higashi-ku, Fukuoka, Japan; Center for Cellular and Molecular Medicine, Kyushu University Hospital, Higashi-ku, Fukuoka, Japan

**Keywords:** IgG4-related dacryoadenitis and sialoadenitis, IgG4-related disease, Marginal zone B cell lymphoma, Mikulicz’s disease, Biopsy

## Abstract

**Background:**

IgG4-related dacryoadenitis and sialoadenitis (IgG4-DS), so-called Mikulicz’s disease, is characterized by elevated serum IgG4 and infiltration of IgG4-positive plasma cells in glandular tissues. Recently, several studies reported both malignant lymphoma developed on the background of IgG4-associated conditions and IgG4-producing malignant lymphoma (non-IgG4-related disease).

**Case presentation:**

We report on the case of a 70-year-old man who was strongly suspected IgG4-DS because of high serum IgG4 concentration (215 mg/dl) and bilateral swelling of parotid and submandibular glands. Biopsies of cervical lymph node and a portion of submandibular gland were performed. These histopathological findings subsequently confirmed a diagnosis of marginal zone B cell lymphoma.

**Conclusion:**

Differential diagnosis of IgG4-DS is necessary from other disorders, including Sjögren’s syndrome, sarcoidosis, Castleman’s disease, Wegener’s granulomatosis, lymphoma, and cancer. We suggest that biopsy of swollen lesions is important for a definitive diagnosis of IgG4-DS and discuss the mechanism of development in this case.

## Background

IgG4-related dacryoadenitis and sialoadenitis (IgG4-DS), also known as Mikulicz’s disease (MD), is a unique condition characterized by enlargement of the lacrimal and salivary glands caused by infiltration of lymphocytes. IgG4-DS has been considered a subtype of Sjögren’s syndrome (SS) because of certain histopathological similarities, particularly lymphocytic infiltration [1]. However, IgG4-DS patients show elevated serum levels of IgG4 and infiltrating IgG4-positive plasma cells in the glandular tissues [2,3]. Similar findings have also been identified in other diseases such as autoimmune pancreatitis [4], interstitial pneumonia [5], retroperitoneal fibrosis [6], and sclerosing cholangitis [7], and these diseases are now referred to as ‘IgG4-related disease (IgG4-RD)’ [8]. IgG4-DS is now diagnosed by both ‘Comprehensive diagnostic criteria for IgG4-related disease (2011)’ and ‘Diagnostic criteria for IgG4-related Mikulicz’s disease D’ approved by the Japanese Society for Sjögren’s syndrome [9]. However, it is important to differentiate IgG4-RD from malignant tumors (such as cancer, lymphoma) and similar diseases (such as SS, primary sclerosing cholangitis, Castleman’s disease, secondary retroperitoneal fibrosis, Wegener’s granulomatosis, sarcoidosis, Churg-Strauss syndrome) by histopathological examination of local lesion. We reported the first case of IgG4-producing marginal zone B cell lymphoma (MZL) in salivary glands mimicking IgG4-DS.

## Case presentation

A 70-year-old man was referred to our institution with bilateral continuous painless swelling of the parotid glands (PGs) and submandibular glands (SMGs) in March 2013. He had previously attended another hospital with chief complaints of dry mouth and bilateral swelling of SMGs, which were diagnosed as chronic sialoadenitis by computed tomography (CT) in January 2013. However, he did not receive any treatment at that time and ignored the swelling. He had no medical history of allergic diseases such as bronchial asthma, atopic dermatitis, or allergic rhinitis.

**Figure 1:**
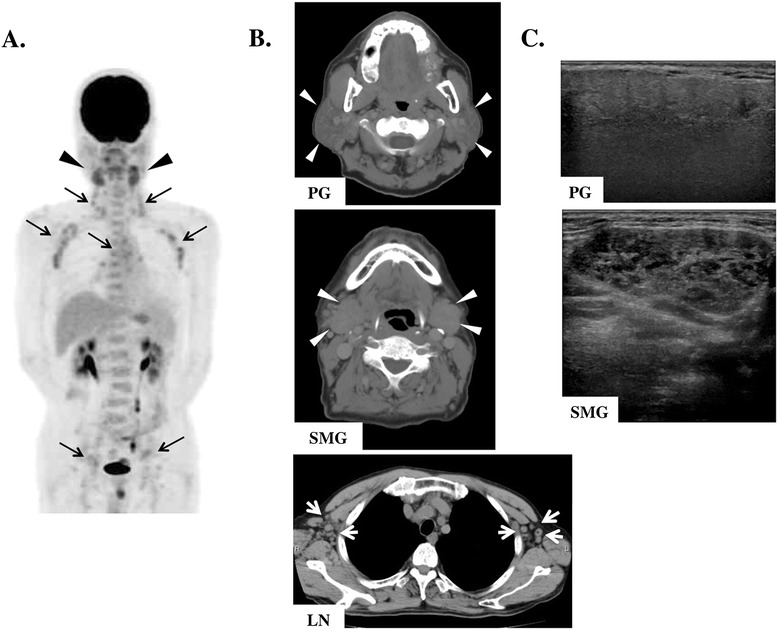
**Imaging findings before treatment. (A)** Fluorodeoxyglucose positron emission tomography (FDG-PET) indicating abnormal multiple accumulations in the submandibular glands (SMGs) (white arrowheads) and in several systemic lymph nodes (black arrowheads). **(B)** Computed tomography (CT) findings indicating swelling of the parotid glands (PGs) (white arrowheads), SMGs (white arrowheads), and generalized lymph nodes (LNs) (white arrows). **(C)** Sonographic images indicating bilateral nodal hypoechoic areas in the SMGs.

Physical findings showed no fever (body temperature, 36.6°C), a blood pressure of 124/68 mmHg, a pulse rate of 64 beats per min, and arterial oxygen saturation of 98% in room air. He had a dry mouth and stomatitis with reduced unstimulated salivary flow rate (0.9 ml/15 min). Bilateral PGs, SMGs, and sublingual glands were elastic, hard, and swollen, but not painful. Several cervical lymph nodes were enlarged. F-18 fluorodeoxyglucose positron emission tomography (FDG-PET) demonstrated abnormal multiple accumulations in the SMGs (SUVmax, 4.63) and in several systemic lymph nodes (SUVmax, 3.23–4.12) (Figure 1A). CT and magnetic resonance imaging showed remarkable swelling of bilateral PGs, SMGs, and generalized lymph nodes, including in the neck (Figure 1b). On ultrasonograms, SMGs showed bilateral nodal hypoechoic areas with high vascularization, whereas PGs showed normal or slight change (Figure 1C).

Laboratory findings are indicated in Table 1. He had an increased erythrocyte sedimentation rate (15 mm/h), hemoglobin concentration of 12.6 g/dl, white blood cell count of 4,680/mm^3^ (neutrophils 40.7%, lymphocytes 49.8%, monocytes 8.5%, eosinophils 0.4%, basophils 0.6%), and platelet count of 12.6 × 10^4^/mm^3^. Serum chemistry data were all within normal limits. His C-reactive protein concentration was 0.07 mg/dl. Immunological tests were negative for anti-nuclear antibody, anti-SS-A antibody, and rheumatoid factor. Serum levels of IgG, IgA, IgM, and IgE were within normal limits (1,177 mg/dl, 179 mg/ml, 91 mg/dl, and 15 IU/ml, respectively), but his serum IgG4 concentration was elevated (215 mg/dl). The proportion of total IgG molecules that were subclass IgG4 was 18%. The serum soluble interelukin-2 receptor (sIL-2R) concentration was abnormally high (1,566.2 U/ml).

**Table 1:** **Laboratory data before and after treatment**

	**Treatment**		**Reference range**
	**Before**	**After**	
WBC (/mm^3^)	4,680	3,620	3,500–9,000
Neutrophils (%)	40.7	-	40.0–70.0
Lymphocytes (%)	49.8	37.0	18.0–53.0
Monocytes (%)	8.5	***12.5***	2.0–12.0
Eosinophils (%)	0.4	2.5	1.0–4.0
Basophils (%)	0.6	***3.0***	0.0–1.0
Hemoglobin (g/dl)	***12.6***	***13.9***	14.0–18.0
Palate (10^4^/mm^3^)	***12.6***	16.6	14.0–44.0
ESR (mm/h)	***12***	-	2–10
Total protein (g/dl)	***6.6***	***6.5***	6.7–8.3
Albumin (g/dl)	4.0	4.3	4.0–5.0
LDH (U/L)	182	177	119–229
CRP (mg/dl)	0.07	0.02	<0.10
IgG (mg/dl)	1,177	993	872–1,815
IgG4 (mg/dl)	***215***	***195***	4.8–105
IgA (mg/dl)	179	196	95–405
IgM (mg/dl)	91	66	31–190
IgE (mg/dl)	15	-	<240
sIL-2R (U/ml)	***1,566.2***	***752.0***	206.0–713.0

*WBC* white blood cell, *ESR* erythrocyte sedimentation rate, *CRP* C-reactive protein, *sIL-2R* soluble interleukin-2 receptor. Bold and italicized numbers indicate abnormal values.

**Figure 2:**
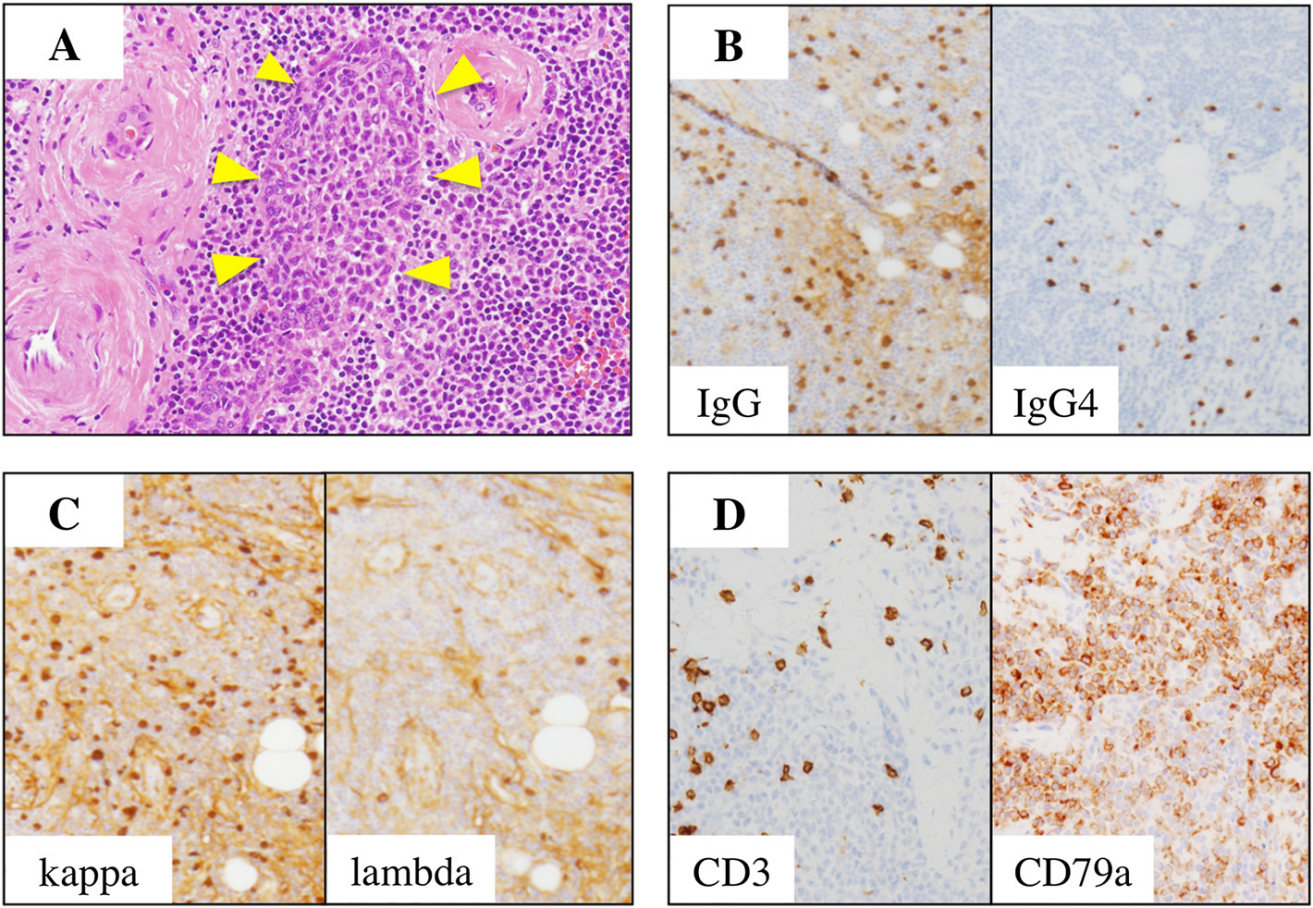
**Histological findings in submandibular glands. (A)** Severe lymphoplasmacytic infiltration with hyperplastic lymphoid follicles, so-called ‘follicular colonization’ (white arrowheads). **(B)** mild infiltration of IgG4-positive plasma cells (IgG4-positive/IgG-positive plasma cell ratio, 10%). **(C)** Monotypic predominance of kappa-light chain. **(D)** Infiltrating lymphocytes were positive for B cell markers (CD79a), but negative for T-cell markers (CD3).

IgG4-DS was initially suspected because of the enlarged glandular tissues and high serum IgG4 concentration. Open biopsies of the cervical lymph node and a portion of the left SMG were therefore performed for definitive diagnosis. Histologically, all sections showed severe lymphoplasmacytic infiltration with hyperplastic lymphoid follicles, so-called follicular colonization. Furthermore, the plasmacytoid cells showed nuclear pleomorphism. Immunohistochemical staining showed mild infiltration of IgG4-positive plasma cells (IgG4-positive/IgG-positive plasma cell ratio, 10%) and monotypic predominance of kappa-light chain. The infiltrating lymphocytes were positive for B cell markers (CD20 and CD79a) and bcl-2, but negative for T-cell markers (CD3, CD5, CD8, and CD45RO), CD10 and cyclinD1 (Figure 2).

**Figure 3:**
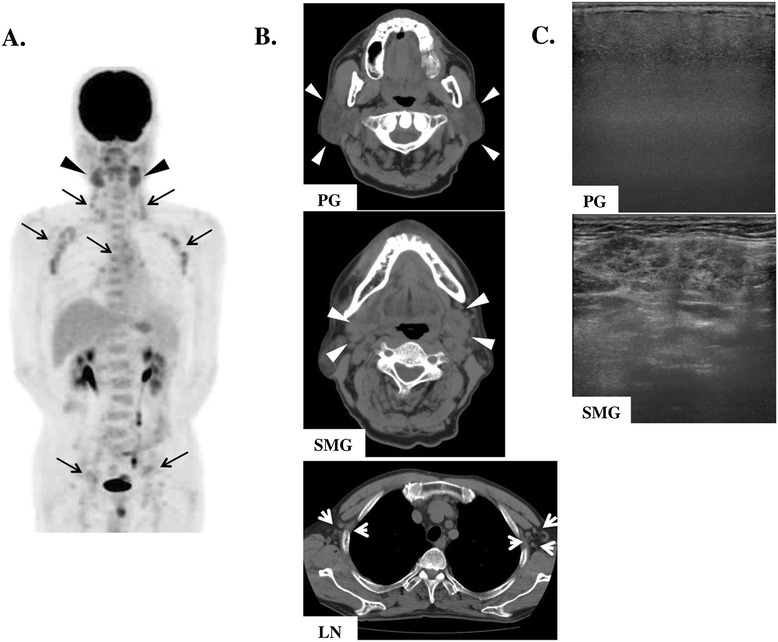
**Imaging findings after treatment.** **(A)** FDG-PET indicating unchanged abnormal multiple accumulations in the SMGs (white arrowheads) and in several systemic lymph nodes (black arrowheads). **(B)** CT findings indicating swelling of the PGs (white arrowheads), SMGs (white arrowheads), and generalized lymph nodes (LNs) (white arrows). **(C)** Sonographic images indicating vaguely-outlined hypoechoic areas in the SMGs.

These histopathological findings and clinical features confirmed a diagnosis of MZL. The patient was treated with 600 mg rituximab for six times as currently recommended regimen [9-11]. Swelling of the SMGs diminished slightly, and there was no metastatic spread. PGs and LNs showed no remarkably change on all images, whereas SMGs had ill-defined borders of nodal hypoechoic areas (Figure 3). Laboratory findings after treatment still revealed high concentrations of serum IgG4 (195 mg/dl) and sIL-2R (752.0 U/ml) (Table 1).

## Conclusions

IgG4-DS is now recognized as a new emerging disorder, characterized by high serum IgG4, marked infiltration of IgG4-positive plasma cells and severe fibrosis with hyperplastic ectopic germinal centers in lacrimal and salivary glands. We recently proposed ‘Comprehensive diagnostic criteria for IgG4-RD’ [12]. IgG4-RD can be diagnosed using these comprehensive diagnostic criteria combined with organ-specific criteria. If a diagnosis of IgG4-DS is probable or possible based on these criteria, it can be confirmed according to the ‘Diagnostic criteria for IgG4-related Mikulicz’s disease’ approved by the Japanese Society for Sjögren’s syndrome in 2008, which include the following items: (i) persistent (longer than 3 months) symmetrical swelling of more than two lacrimal and major salivary glands, (ii) raised serum levels of IgG4 (>135 mg/dl), and (iii) infiltration of IgG4-positive plasma cells in the tissue (IgG4-positive plasma cells/IgG-positive plasma cells >0.4) by immunostaining. For a positive IgG4-DS diagnosis, any two of these three criteria must be fulfilled, including item (i). The present case met criteria (i) and (ii), and IgG4-DS was therefore strongly suspected. However, biopsy of the local lesion is recommended for differential diagnosis from other disorders, including sarcoidosis, Castleman’s disease, Wegener’s granulomatosis, lymphoma, and cancer. We therefore performed the cervical lymph node and SMG biopsies, resulting in a definitive diagnosis of MZL. These results suggest that biopsy of the swollen lesion is essential for a definitive diagnosis of IgG4-DS. Moreover, we have commonly performed incisional open biopsies of swollen SMGs under local anesthesia, with no complications including facial palsy, sialoceles, or wound infections. Open biopsy is thus a relatively low-invasive procedure and useful for the definitive diagnosis of IgG4-DS [13].

Isabel et al. [14] reported IgG4 expression in MZLs of various primary sites. According to their paper, 19 out of 49 (38.8%) cutaneous MZLs were positive for IgG4 expression, while only 1 out of 120 (0.8%) other MZLs were positive, especially all of 20 salivary gland MZLs showed no IgG4 expression. Therefore, salivary gland MZL positive for IgG4 as in this case is very rare. Several studies have reported IgG4-associated conditions in parts of malignant lymphoma of the ocular adnexal and lymph nodes [15]. However, IgG4-positive cells were non-neoplastic, and this IgG4-related chronic inflammation is thought to be a background for the development of malignant lymphoma [16,17]. In contrast, IgG4-producing lymphoma (non-associated IgG4-RD) also exists [18], in which the IgG4-producing cells are neoplastic and monoclonal rearrangement bands can be observed by Southern blot hybridization.

Single-agent therapy of rituximab is not effective for MZL but for IgG4-RD [19]. In this case, the clinical findings, images, and laboratory examinations showed no remarkably improvement after treatment for rituximab for six times. His serum IgG4 concentration levels remained high and swelling of salivary glands was nearly unchanged, which indicated that this case might be IgG4-producing MZL but not on a background of IgG4-related chronic inflammation. Unfortunately, we were unable to confirm a strong infiltration of IgG4-positive cells in the biopsy specimens, and more case reports and further examinations are required to clarify the clinicopathological features of IgG4-RD.

## Consent

Written informed consent was obtained from the patient for the publication of this report and any accompanying images.

## Abbreviations

IgG4-DS: IgG4-related dacryoadenitis and sialoadenitis
MD: Mikulicz’s disease
SS: Sjögren’s syndrome
IgG4-RD: IgG4-related disease
MZL: marginal zone B cell lymphoma
PG: parotid gland
SMG: submandibular gland
CT: computed tomography
FDG-PET: F-18 fluorodeoxyglucose positron emission tomography
